# Various Configurations for Improving the Efficiency of Metallic and Superconducting Photocathodes Prepared by Pulsed Laser Deposition: A Comparative Review

**DOI:** 10.3390/ma17215257

**Published:** 2024-10-29

**Authors:** Alessio Perrone, Muhammad Rizwan Aziz, Francisco Gontad

**Affiliations:** 1 Dipartimento di Matematica e Fisica “E. De Giorgi”, Università del Salento, 73100 Lecce, Italy; alessio.perrone@unisalento.it; 2 INFN-Istituto Nazionale di Fisica Nucleare, 73100 Lecce, Italy; 3 Laser Applications Centre, Asociación de Investigación Metalúrgica del Noroeste, 36410 Porriño, Spain; francisco.gontad@aimen.es

**Keywords:** quantum efficiency, pulsed-laser deposition, thin films, metallic and superconducting photocathodes, photocathode configuration

## Abstract

This paper presents an innovative exploration of advanced configurations for enhancing the efficiency of metallic and superconducting photocathodes (MPs and SCPs) produced via pulsed laser deposition (PLD). These photocathodes are critical for driving next-generation free-electron lasers (FELs) and plasma-based accelerators, both of which demand electron sources with improved quantum efficiency (QE) and electrical properties. Our approach compares three distinct photocathode configurations, namely: conventional, hybrid, and non-conventional, focusing on recent innovations. Hybrid MPs integrate a thin, high-performance, photo-emissive film, often yttrium or magnesium, positioned centrally on the copper flange of the photo-injector. For hybrid SCPs, a thin film of lead is used, offering a higher quantum efficiency than niobium bulk. This study also introduces non-conventional configurations, such as yttrium and lead disks partially coated with copper and niobium films, respectively. These designs utilize the unique properties of each material to achieve enhanced photoemission and long-term stability. The novelty of this approach lies in leveraging the advantages of bulk photoemission materials like yttrium and lead, while maintaining the electrical compatibility and durability required for integration into RF cavities. The findings highlight the potential of these configurations to significantly outperform traditional photocathodes, offering higher QE and extended operational lifetimes. This comparative analysis provides new insights into the fabrication of high-efficiency photocathodes, setting the foundation for future advancements in electron source technologies.

## 1. Introduction

**Figure 1:**
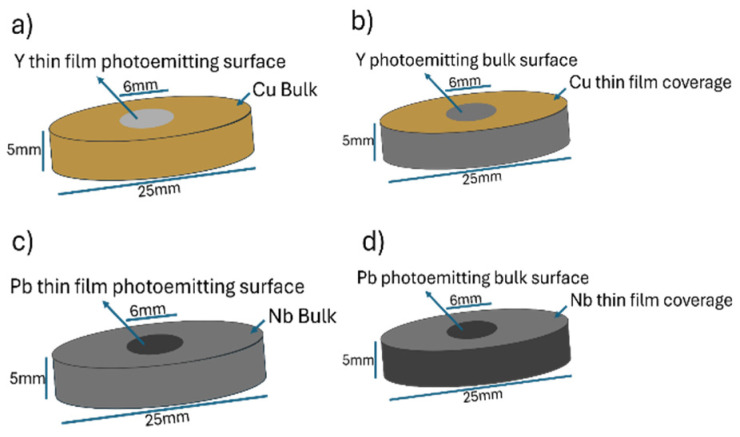
Scheme of the hybrid and non-conventional configurations for MP (**a**,**b**) and for SCP (**c**,**d**), respectively.

Research into traditional metallic photocathodes (MPs) and superconducting photocathodes (SCPs) has attracted considerable attention due to their ability to generate high-brightness electron beams, their fast photoemission, which leads to a low temporal spread, the larger availability of electrons leading to the shortest time between electron pulses, or better electrical coupling with metallic cavities [1,2,3,4,5]. These technologies play crucial roles in the latest X-ray spectrum free-electron lasers (FELs) and are pivotal for advancements in particle-driven plasma wakefield accelerators [6,7,8,9,10,11]. The ongoing demand for electron beams characterized by high peak currents and minimal dark emittance has prompted researchers to explore alternative materials, moving beyond the conventional use of copper (Cu) and niobium (Nb) in Normal-Conducting Radio Frequency (NCRF) and Superconducting Radio Frequency (SRF) guns [12,13,14,15,16]. Recent years have seen progress in hybrid MPs, particularly those created by pulsed-laser deposition (PLD) on Cu substrates with yttrium (Y) and, to a lesser extent, magnesium (Mg) thin films. These metals have demonstrated higher quantum efficiency (QE) compared to Cu, yielding promising results [17,18,19,20,21]. Therefore, we have studied and developed new photocathode layouts called “hybrid configuration”, based mainly on thin films of Mg or Y [18,21]. In the hybrid configuration depicted in Figure 1a, a layer of thin metal film is applied to the central area of the substrate using PLD. These films have shown strong adherence and a satisfactory photoemission performance, evidenced by their QE [17,22]. However, the laser cleaning process necessary before the photoemission effect reduces the thickness of these already thin films (0.1–2 µm), significantly shortening their lifespan [3,23,24].

To address this issue, another novel configuration has been introduced [24]. Here, the thin film deposited by PLD is designed to improve the bulk material’s photoemission and enhance the electrical compatibility with the RF-Gun cavity. This new MP prototype features a Y disk (25 mm diameter, 5 mm thickness) with a thin Cu film, leaving a 6 mm middle circular section of the Y substrate exposed as the emitting area, as shown in Figure 1b. This configuration allows the photoemission to occur directly from the Y bulk, while the RF gun cavity interacts with the surrounding Cu surface. This engineered setup integrates the advantageous combination of the high QE and low work function (WF) of Y with the necessary electrical compatibility suitable for Cu-based NCRF guns.

An analogous approach has been suggested for SCPs [25]. While pure Nb can function as a photocathode material within Nb SRF guns, its low QE limits the electron beam intensity [2]. Efforts to enhance SCP performance have included covering the Nb bulk with a thin lead (Pb) film [15], which is known as hybrid configurations which have also been explored, as illustrated in Figure 1c. However, Pb films tend to oxidize when exposed to air, which, although less severe than other semiconductor photocathodes, still poses a challenge [26,27]. Like MPs, the main drawback of hybrid SCPs is the thinning of materials due to the laser cleaning required to rejuvenate the emissive surface. To mitigate this, a similar innovative configuration has been proposed for SCPs. This involves a Pb substrate coated by a thin Nb film, with the central area masked to serve as the electron source (see Figure 1d). This design maintains the cavity’s Q-value and does not compromise the applicable electric fields. These special configurations are not necessary to develop semiconductor photocathodes, such as gallium arsenide (GaAs) and cesium telluride (Cs_2_Te) for their superior QE compared to traditional metal photocathodes. GaAs, in particular, is notable for its negative electron affinity surface, which dramatically enhances QE when activated with cesium and oxygen [28,29]. However, these materials often suffer from rapid degradation due to chemical reactions with residual gases in the vacuum system, which necessitates frequent reactivation [30]. Furthermore, the operational lifespan of semiconductor photocathodes can be limited by the damage incurred during the high-intensity laser cleaning process, similar to the challenges faced with metallic and superconducting counterparts [30]. Advanced techniques, such as atomic layer deposition (ALD) and molecular beam epitaxy (MBE), are being explored to create more robust and long-lasting semiconductor photocathodes by precisely controlling the film thickness and composition. 

In this paper, we present our findings on MPs and SCPs, focusing on the non-conventional configurations developed to enhance the photoemission performance and extend their operational lifespan. The advantages and disadvantages of the three different photocathode configurations are reported in Table 1.

## 2. The Choice of the Deposition Technique

Various techniques have been employed to deposit metallic thin films used as electron sources [15,31,32]. Among these methods, the PLD technique stands out for its ability to ensure the robust bonding of metallic films to substrates, even when applied at an ambient temperature [21,32,33]. When selecting a deposition technique for metals such as Cu, Mg, and Pb for photocathode applications, the PLD technique presents distinct advantages over other Physical Vapor Deposition (PVD) methods. Techniques such as thermal evaporation, electron beam evaporation, sputter deposition, cathodic arc deposition, and molecular beam epitaxy (MBE) each have specific drawbacks that make them less suitable for high-quality photocathode fabrication.

Thermal evaporation often suffers from contamination due to the inclusion of impurities in the evaporated material. Additionally, achieving precise control over the film thickness is challenging, and scaling the process for larger substrates is problematic [34]. Moreover, the adherence of the film deposited by this technique is poor [31]. Electron beam evaporation, while offering some improvements, introduces high equipment costs and substrate heating issues that can damage sensitive materials. This method also struggles with precise thickness control, particularly for materials with high melting points [35].

Sputter deposition, another common PVD technique, frequently induces stress in the films, leading to potential cracking or delamination. Its lower deposition rates extend the process times, and the high-energy ions used can damage the substrate [36]. Cathodic arc deposition, although capable of high deposition rates, produces droplets that result in rough surfaces and film defects. This method also causes significant substrate heating and potential contamination from the cathode material [34].

MBE, known for producing high-quality films, is hindered by extremely high costs, slow deposition rates, and the requirement for ultra-high vacuum conditions. These factors increase the operational complexity and restrict the throughput, making MBE less suitable for large-scale applications [37].

In contrast, PLD offers numerous advantages. It can produce high-quality, uniform thin films with smooth surfaces and provides precise control of the deposited films’ thickness. PLD is versatile, capable of depositing a wide range of materials, including complex alloys and compounds, while minimizing contamination due to the laser ablation process. Despite its relatively low deposition rates, it is suitable for thin films, and can be optimized to minimize substrate heating and damage. These attributes make PLD a superior choice for the deposition of metals like Cu, Mg, and Pb for photocathode applications [38].

A common setup for PLD involves a high vacuum chamber made of stainless steel (10^−5^–10^−6^ Pa), where the substrate is placed parallel to the target, typically 4–5 cm apart. To prevent the development of significant craters or directional formations, the target undergoes rotation and displacement during deposition, a practice followed in our previous research. Ultraviolet-visible lasers are frequently utilized due to their effective interaction with various target materials. The pulsed laser beam strikes the target surface at a 45° angle, delivering a power density ranging from 0.1 to 1 GW/cm^2^, within nanosecond durations. Depending on specific parameters, metallic films are deposited on the substrate at rates typically measured in tenths of an angstrom per laser pulse. A notable strength of PLD lies in the strong adherence of these films to the substrate, a characteristic that has been consistently highlighted. Measurements using time-of-flight techniques indicate that the plasma’s average ion kinetic energy can vary significantly, potentially reaching several hundred electron volts, depending on the laser intensity [39,40,41,42]. The QE of the films is typically measured in a high vacuum system where the photocathode is aligned in front of the anode such that a laser beam can be focused on the photocathode surface without affecting the anode. An intense electric field, within the megavolt per meter range, is applied between the anode and cathode, and the emitted current is measured with respect to the laser energy hitting the photocathode surface. The QE of the photocathodes prepared by PLD was studied using a custom-built photodiode cell, designed according to the specifications mentioned earlier, as illustrated in Ref. [31].

## 3. Photocathodes Assembly

### 3.1. Hybrid and Non-Conventional Configurations of Metallic Photocathodes

**Figure 2:**
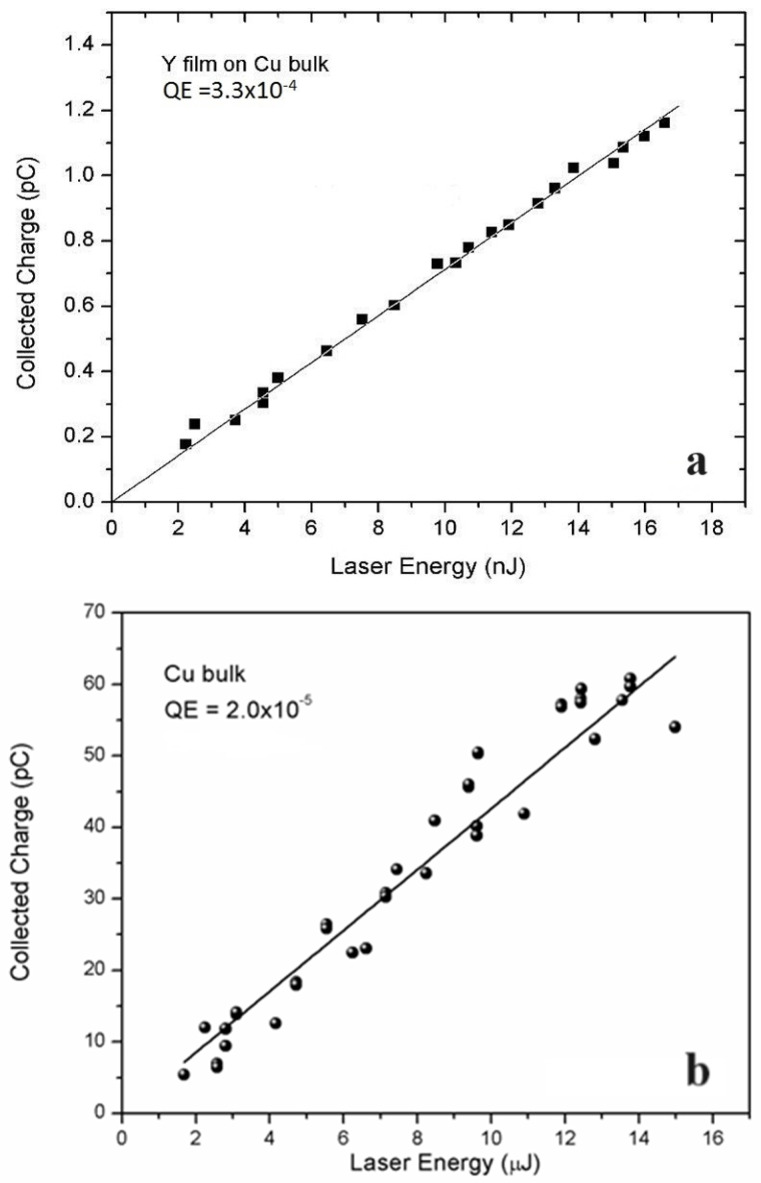
Collected charge as a function of laser energy after laser cleaning treatment for Y thin film deposited on Cu in hybrid configuration (**a**) and for Cu bulk (**b**). Continuous lines are the data-fitting curves (Figure 2a reproduced from ref. [21] and Figure 2b reproduced from ref. [17]).

Previous studies extensively cover the operational principles and characteristics of traditional MPs [3,19,43]. Copper is frequently used in these applications due to its high chemical stability, long lifespan, low electrical resistivity, good thermal conductivity and economical cost, which enhance the quality factor of RF cavities. Nevertheless, Cu exhibits a low QE of 2.0 × 10^−5^ at 266 nm [17], necessitating the utilization of powerful UV lasers to overcome its high WF (see Table 2). Even photocathodes based on Mg with a WF of 3.6 eV require the use of the fourth harmonic of a Nd:YAG laser (266 nm, 4.66 eV) or the third harmonic of a Ti:sapphire (267 nm, 4.64 eV). That means much less energy and stability of the drive laser. On the contrary, the low WF value of Y (3.1 eV) allows one to drive photocathodes based on this metal with the third harmonic (355 nm, 3.5 eV) of the Nd:YAG laser. However, many facilities still use Mg [44] as a photocathode due to its relatively high QE (7.6 × 10^−4^ at 266 nm) and to the lower chemical reactivity than that of Y. These two metals react with hydrogen- and oxygen-containing molecules such as H_2_, H_2_O, CO_2_, and O_2_, forming hydrides and oxides on the surface [45]. However, it is important to note that the oxidation processes in bulk metal photocathodes are significantly less severe compared to those in semiconductor photocathodes. Recently, “smart” cathodes designed with thin films to enhance QE and extend longevity have been developed. The hybrid design, featuring a thin film realized on the middle part of a conventional metallic cathode (see Figure 1a,c), has been implemented for both MPs and SCPs. Figure 2a,b illustrate the collected charge vs. the function of laser energy for Y thin films on Cu in a hybrid configuration and for Cu bulk after laser cleaning. The linear correlation between the collected charge and laser energy suggests that photoemission takes place through a one-photon absorption mechanism. Additionally, the minimal space charge effect, resulting from the low collected charge, further supports this. The slope corresponding to the continuous line correlates to the QE value [32]. Figure 2a,b illustrate that the QE of the Y thin film on Cu is substantially greater than that of bulk Cu under identical conditions and is also corroborated by the literature [21]. The most important photo-emissive properties of the metals used in metallic and superconducting photocathodes are shown in Table 2.

As previously noted, Y and Mg films deposited by the PLD technique typically range from hundreds of nanometers to a few micrometers in thickness, often insufficient to endure repeated cleaning processes required for operational use. Thicker films, whether deposited by PLD or other methods, often result in high mechanical strain and cracking, leading to the detachment of the film from the RF gun back flange. Several approaches have been suggested to resolve this problem, including friction welding [52], hot isostatic pressing [53] and press-fitting bulk disks [47].

However, inserting a disk of another material into the middle section of a Cu photocathode introduces minute discontinuities between the two metals, causing undesirable electrical discharges and reducing the RF cavity’s quality factor. On the contrary, the discontinuity between the substrate and the thin film in the non-conventional layout is very smooth. Thus, the non-conventional configuration mentioned earlier and shown in Figure 1b,d appears to be the most effective alternative to prevent these issues and the thinning process [48]. This innovative design allows the metallic photocathode to retain the photoemissive properties of Y bulk [24]. Indeed, with the present assembly, the laser cleaning process is related to bulk metal (Y or Mg) and not to the thin film. The most thoroughly investigated MP in this non-conventional setup is a Cu thin film annularly realized on a Y disk (see Figure 1b), combining photoemissive characteristics of Y with electrical properties of Cu. The conductive Cu thin films were analyzed morphologically, structurally and electrically to ensure their suitability for application. Scanning electron microscopy (SEM) showed the presence of small droplets on the Cu surface, albeit in smaller quantities compared to other metal deposition techniques [2].

**Figure 3:**
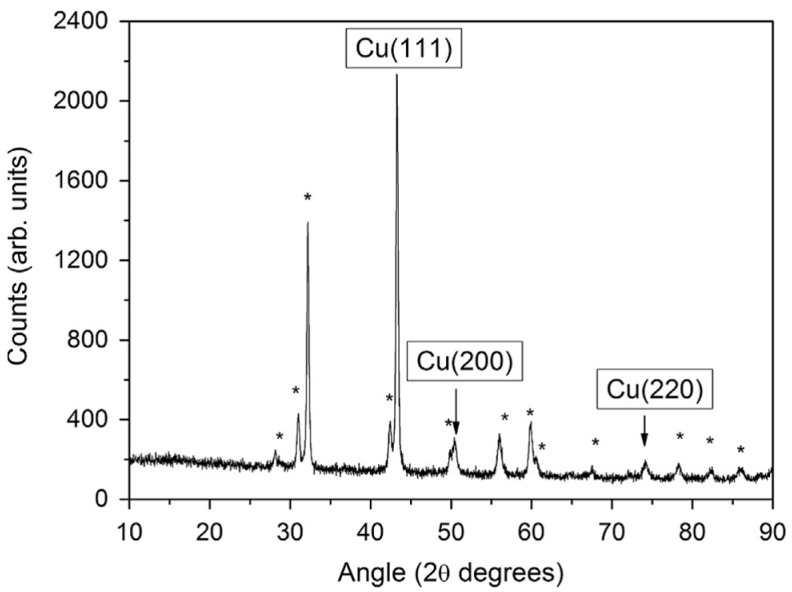
XRD pattern of Cu film deposited by PLD technique on a Y disk by sub-ps laser pulses. The peaks labeled as * can be ascribed to the Y polycrystalline (reproduced from ref. [48]).

The structural characteristics of the Cu film on the Y substrate, deposited by sub-ps laser pulses, utilized as a photocathode, were analyzed by X-ray diffraction (XRD). The XRD pattern in Figure 3 indicates peaks corresponding to the (111), (200) and (220) crystallographic planes of Cu, while other peaks are attributed to the Y polycrystalline substrate. Both morphological and structural analyses indicate the Cu film’s promising properties for application, and its high adherence to the Y substrate is also confirmed.

This excellent physical performance is mainly tied to the substantial kinetic energy of particles within the Cu plasma during the laser ablation procedure. The mean electrical resistivity of a 100 nm thick Cu film on a Y substrate was measured at 2.3 × 10^−6^ Ω·cm, translating to a sheet resistance of 3.1 × 10^−1^ Ω/sq [24]. This measured resistivity value is about 36% higher than that of bulk high-purity Cu (1.69 × 10^−6^ Ω × cm) [54], likely due to electron scattering caused by the atomic-scale surface roughness of the deposited film [55]. 

### 3.2. Hybrid and Non-Conventional Configurations of Superconducting Photocathodes

**Figure 4:**
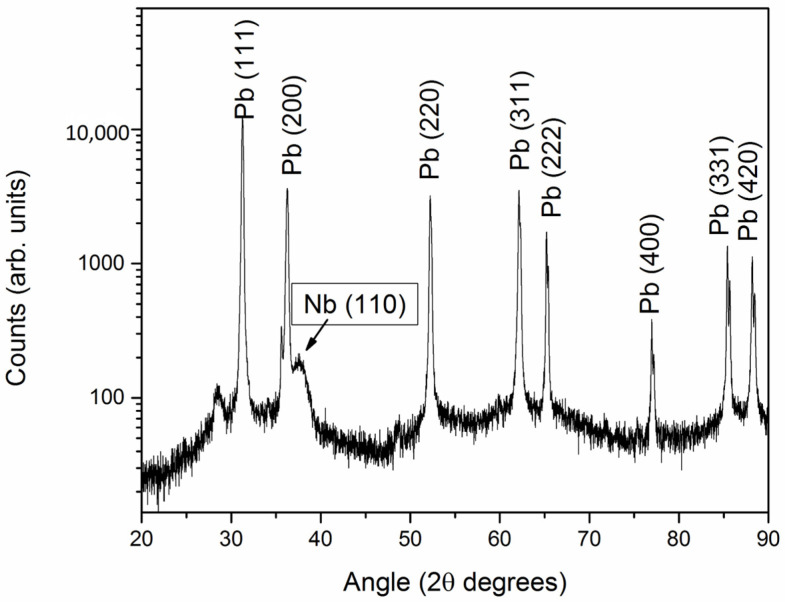
XRD pattern of the Nb PLD film deposited on Pb substrate (reproduced from ref. [25]).

Superconducting photocathodes leverage the unique properties of superconducting materials to generate high-brightness electron beams with minimal energy dissipation. Nb and Pb are the most commonly used materials in SCPs due to their favorable superconducting properties. Due to its high critical temperature and good mechanical properties, is widely used in SRF cavities. Pb, although having a lower critical temperature, offers a higher QE when used as a photoemissive material. The combination of these materials in hybrid configurations aims to utilize the high QE of Pb and the robust superconducting characteristics of Nb [25]. The deposition methods, like PLD, for creating these hybrid configurations are critical as they affect the film’s adherence, uniformity, and overall performance. The challenge of maintaining surface cleanliness and preventing oxidation is paramount, as these factors directly impact the photoemissive efficiency and lifespan of SCPs. The hybrid configuration for superconducting photocathodes (SCPs) primarily incorporates Nb and Pb metals. These devices are created by depositing a thin Pb film onto a Nb substrate employing numerous deposition methods, such as electroplating, arc deposition, sputtering, and thermal evaporation [15,31,34,56,57,58]. Figure 4 shows the XRD pattern of the Nb film, indicating that the PLD film is largely amorphous, with a weak peak around 38° corresponding to the (110) crystalline planes. Other peaks are attributed to the Pb substrate. 

**Figure 5:**
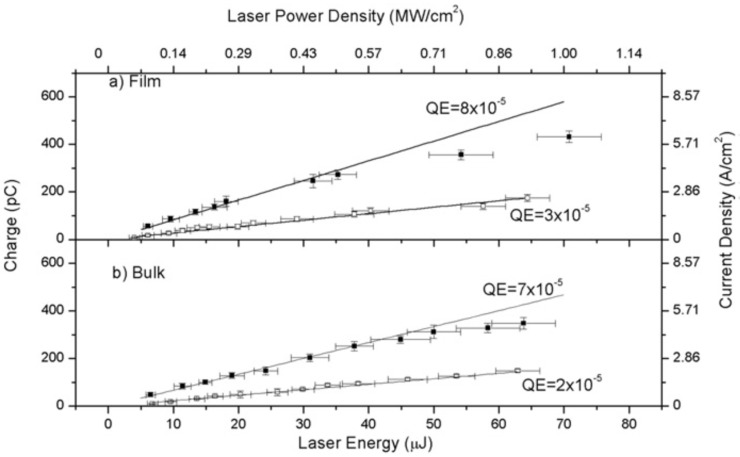
Charge of the electrons emitted from (**a**) Pb film prepared by PLD in hybrid configuration and (**b**) Pb bulk before (□) and after (■) laser cleaning treatment (reproduced from ref. [50]).

Figure 5a,b display the electron charge emitted from a Pb thin film prepared by PLD in a hybrid configuration compared to Pb bulk versus the laser energy.

The QE values for Pb bulk and Pb thin film are 7 × 10^−5^ and 8 × 10^−5^ at 266 nm, respectively [50], which are significantly higher than the 3.2 × 10^−6^ QE of the Nb bulk at 248 nm [13]. However, it is observed that a slight space charge effect occurs at laser energies exceeding 35 μJ. Despite the good chemical stability of Pb, its surface oxidizes when exposed to air, necessitating laser cleaning before its use in SRF guns. As with MPs, these cleaning processes lead to the thinning of the photoemitting surface, compromising the performance and lifespan. Consequently, alternative mechanical and physical solutions have been explored by various research groups.

In a non-conventional set-up, a configuration was proposed where a Pb disk is entirely coated with a Nb thin film, excluding its central surface, which serves as the photo-emitting area (Figure 1d) [25]. As in the case of metallic photocathodes, in the non-conventional configuration the drive laser acts on the Pb bulk and not on the thin film. Mechanical solutions face similar challenges as those with MPs; thus, PLD appears to be an optimal technique for depositing highly adherent thin films. The Nb films were analyzed for their morphological, structural and electrical properties, as well as mechanical properties.

Nano-indentation analysis determined the mechanical properties of the Nb film, revealing a good hardness value of 2.8 ± 0.3 GPa [25]. It is crucial to avoid surface scratches on photocathodes, as these can cause electrical discharges under the high electric fields in superconducting cavities.

**Figure 6:**
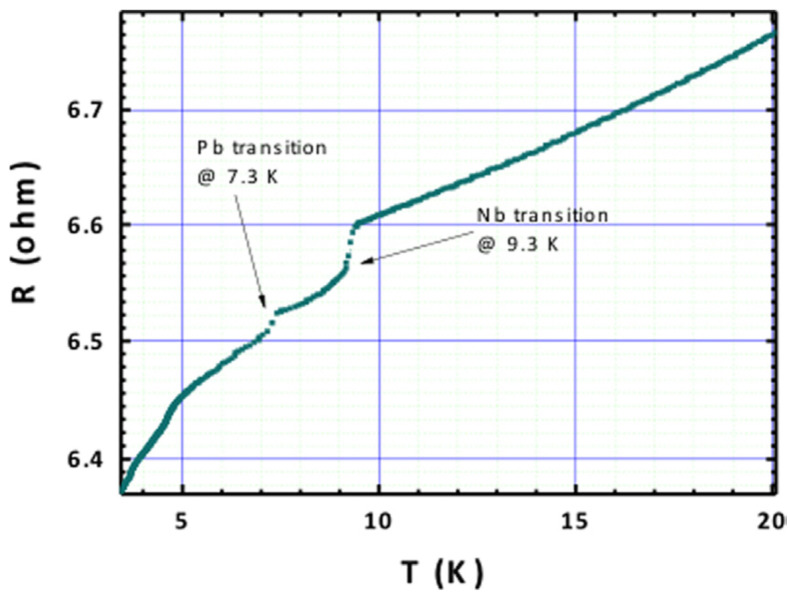
Electrical resistance evolution of the sample Nb thin film on Pb substrate with the temperature (reproduced from ref. [25]).

Another vital characteristic of SCPs is their electrical compatibility with superconducting cavities. Figure 6 shows the sample resistance as a function of temperature, with two noticeable discontinuities: one at 9.3 K, corresponding to the critical temperature of Nb, and another at 7.3 K, near the critical temperature of Pb [25].

In this non-conventional configuration, SCPs have similar photoelectrical properties to Pb bulk. These findings indicate that the non-conventional setups for both MPs and SCPs show the greatest potential and dependability among the various proposed configurations (see Table 1, Table 2, Table 3 and Table 4).

## 4. Conclusions

This study presents a comprehensive exploration of advanced photocathode configurations aimed at enhancing the performance of MPs and SCPs fabricated via PLD. The comparative analysis of conventional, hybrid, and non-conventional configurations demonstrates significant improvements in QE, operational longevity, and integration with RF cavities.

The hybrid configuration, where thin films of high-performance materials such as Y or Pb are deposited onto conventional Cu or Nb substrates, offers distinct advantages. This approach enhances the photoemission properties while maintaining good electrical compatibility with RF cavities. The thin film design allows for a higher QE than conventional metallic photocathodes, making it a viable solution for improving the efficiency of electron sources. However, the hybrid configuration faces challenges related to the thinning of the photoemissive layer during the laser cleaning process, which limits its operational lifespan. Despite this, the hybrid photocathodes represent a significant step forward in leveraging thin-film technology to optimize the photocathode performance.

In parallel, the non-conventional configuration represents a further advancement by mitigating the thinning issue. By using bulk Y and Pb materials directly exposed to the drive laser, while surrounding these with Cu or Nb films for electrical compatibility, this configuration combines the best of both worlds: the superior photoemission of Y and Pb and the robust structural and electrical properties of Cu and Nb. This design not only extends the operational lifespan, but also maintains a high QE over time, positioning the non-conventional configuration as a highly durable and efficient option for demanding applications. Both the hybrid and non-conventional configurations underscore the novelty of this study, as they provide new pathways for improving the photoemission efficiency and stability of photocathodes used in next-generation accelerators such as FELs and plasma wakefield accelerators. The hybrid configuration offers a near-term solution with a higher QE than conventional designs, while the non-conventional configuration pushes the boundaries of photocathode technology by addressing long-term operational issues.

These findings lay the groundwork for future innovations in photocathode design, demonstrating the potential for integrating advanced materials and configurations to enhance the electron source performance. The contributions of both configurations represent substantial progress toward meeting the stringent requirements of modern accelerator technologies.

## Figures and Tables

**Table 1:** Advantages and disadvantages of the different photocathode configurations.

Configuration	MP	SCP	Lifetime	QE
Conventional	Cu disk	Nb disk	Relatively long ^1^	Relatively small ^2^
Hybrid	Cu disk with a Y thin film deposited on the central area	Nb disk with a Pb thin film deposited on the central area	Shorter than conventional ^3^	Higher than conventional ^4^
Non-conventional	Y disk with a Cu annular thin film	Pb disk with a Nb annular thin film	Much higher than hybrid ^5^	Higher than conventional ^4^

^1^ High chemical inertness; ^2^ High WF; ^3^ Thinning of the film due to the laser cleaning treatment; ^4^ Low WF; ^5^ Bulk as photoelectron source.

**Table 2:** Relevant photo-emissive properties of the most used bulk metallic photocathodes (conventional configuration).

	Cu	Mg	Y	Nb	Pb
Work function (eV)	4.6 [46]	3.6 [47]	3.1 [48]	4.3 [49]	4.2 [49]
QE (at 266 nm) Bulk	2.0 × 10^−5^ [17]	7.6 × 10^−4^ [21]	3.0 × 10^−4^ [21]	3.2 × 10^−6^ at 248 nm [13]	7 × 10^−5^ [50]
Operational lifetime (yrs) [51]	No limit	~1	<1	No limit	>1
Compatibility with a Cu RF gun	Very high	Medium	Low	-	-
Compatibility with a Nb SCRF gun	-	-	-	Very high	High

**Table 3:** Photo-emissive properties of PLD thin film in hybrid configuration.

	Mg	Y	Pb
QE (at 266 nm)	1.8 × 10^−3^ [21]	3.3 × 10^−4^ [21]	8.0 × 10^−5^ [50]
Operational lifetime *	2–3 months [18]	3–5 months *	4–6 months [31]
Compatibility with a Cu RF gun	High	Medium	-
Compatibility with a Nb SCRF gun	-	-	High

* Expected value.

**Table 4:** Photo-emissive properties of PLD thin film in non-conventional configuration.

	Mg Bulk Cathode with Cu-Mask	Y Bulk Cathode with Cu-Mask	Pb Bulk Cathode with Nb-Mask
QE (at 266 nm)	7.6 × 10^−4^	3 × 10^−4^	7 × 10^−5^ [50]
Operational lifetime (yrs) Similar to those of bulk materials given in Table 2	~1	<1	>1
Compatibility with a Cu RF gun	Very high	High	-
Compatibility with a Nb SCRF gun	-	-	Very high
